# An Efficient Antioxidant System in a Long-Lived Termite Queen

**DOI:** 10.1371/journal.pone.0167412

**Published:** 2017-01-11

**Authors:** Eisuke Tasaki, Kazuya Kobayashi, Kenji Matsuura, Yoshihito Iuchi

**Affiliations:** 1 Department of Applied Bioresources Chemistry, The United Graduate School of Agriculture, Tottori University, Tottori, Japan; 2 Department of Biological Chemistry, Faculty of Agriculture, Yamaguchi University, Yamaguchi, Japan; 3 Department of Applied Biosciences, Graduate School of Agriculture, Kyoto University, Kyoto, Japan; 4 Graduate School of Sciences and Technology for Innovation, Yamaguchi University, Yamaguchi, Japan; University of North Carolina at Greensboro, UNITED STATES

## Abstract

The trade-off between reproduction and longevity is known in wide variety of animals. Social insect queens are rare organisms that can achieve a long lifespan without sacrificing fecundity. The extended longevity of social insect queens, which contradicts the trade-off, has attracted much attention because it implies the existence of an extraordinary anti-aging mechanism. Here, we show that queens of the termite *Reticulitermes speratus* incur significantly lower oxidative damage to DNA, protein and lipid and have higher activity of antioxidant enzymes than non-reproductive individuals (workers and soldiers). The levels of 8-hydroxy-2’-deoxyguanosine (oxidative damage marker of DNA) were lower in queens than in workers after UV irradiation. Queens also showed lower levels of protein carbonyls and malondialdehyde (oxidative damage markers of protein and lipid, respectively). The antioxidant enzymes of insects are generally composed of catalase (CAT) and peroxiredoxin (Prx). Queens showed more than two times higher CAT activity and more than seven times higher expression levels of the CAT gene *RsCAT1* than workers. The CAT activity of termite queens was also markedly higher in comparison with other solitary insects and the queens of eusocial Hymenoptera. In addition, queens showed higher expression levels of the Prx gene *RsPRX6*. These results suggested that this efficient antioxidant system can partly explain why termite queens achieve long life. This study provides important insights into the evolutionary linkage of reproductive division of labor and the development of queens’ oxidative stress resistance in social insects.

## Introduction

The key character of eusociality is reproductive division of labor within collaborative groups. Social species such as ants, honeybees, and termites have a one or a limited number of individuals that produce most or all of the offspring (queens), and a large number of individuals that forego reproduction for group beneficial activities (workers). In these insects, queens live up to 10 times longer than non-reproductive workers [1–4]. Longevity is typically negatively correlated with fecundity and the extent of this trade-off varies within and among species [5]. Previous studies have shown that germline-ablated worms had an extended lifespan [6], and sterile females also showed greater longevity compared with fertile flies [7]. Although most animal species show a gradual decline in reproduction with age [8], social insect queens are thought to be the only animals known that can live for long periods while also producing many offspring per day [9]. Because of their abnormal characteristics implying the presence of an extraordinary anti-aging mechanism, social insect queens have attracted much attention, and they are promising subjects for aging research [10]. However, the molecular mechanisms that allow social insects queens to have great longevity are not yet understood.

The oxidative stress theory of aging states that the accumulation of oxidative damage causes aging [11]. Reactive oxygen species (ROS), typically caused by environment stress, aerobic metabolism, and reproduction, play a positive role in processes such as cell growth signaling at permissible levels, but over-generation cause injurious oxidative stress to biomolecules and the accumulation of damage is associated with aging and negative effects on longevity [12–15]. Several enzymes such as catalase (CAT) and peroxiredoxin (Prx) are involved in ROS detoxification. Hydrogen peroxide (H_2_O_2_) can transform into a highly reactive hydroxyl radical in the presence of reduced metal atoms. CAT efficiently converts H_2_O_2_ to water and oxygen without the production of other ROS. Prx also reduces H_2_O_2_ and functions only when coupled to a sulfhydryl-reducing system such as thioredoxin or glutathione. These antioxidant enzyme activities contribute to stress resistance associated with an organism’s lifespan. Treatment with CAT and superoxide dismutase (SOD) mimetics extended longevity because of the protective effect against oxidative stress in *Caenorhabditis elegans* [16,17]. In the model insect *Drosophila melanogaster*, overexpression of CAT and SOD resulted in reduced levels of oxidative stress and an extended lifespan [18, 19]. Therefore, long-lived social insect queens should have efficient antioxidant systems that eliminate ROS more effectively in order to prevent the accumulation of oxidative damage, in part due to high fecundity [20, 21]. In relation to the hypothesis that antioxidant activity mediates longevity of social insect queens, several reports have been published. Parker et al. showed that copper-zinc SOD (Cu/Zn-SOD) activity and SOD gene expression do not associate with long lifespan of queens in the black garden ant *Lasius niger* [22]. Corona et al., who also obtained similar results, demonstrated that honeybee *Apis mellifera* queens have lower or equal levels of antioxidant gene expression in comparison with workers [23]. Importantly, these studies indicated that a robust antioxidant activity is not prerequisite for longevity in social insect queens and is confined to only eusocial Hymenoptera (ants and honeybees). Eusocial Isoptera (termite) queens may also have as long a lifespan and higher fecundity as the queens of Hymenoptera [2]; however, termite queens have never been studied. Therefore, we focused on a subterranean termite *Reticulitermes speratus* and paid attention to their antioxidant system against oxidative stress.

In this study, we investigated whether long-lived and fertile termite queens have higher antioxidant activities than non-reproductive individuals. First, we found that the oxidation levels of DNA, protein, and lipid were significantly lower in queens of *R*. *speratus* in comparison with workers. To our knowledge, this is first report about the differences in oxidative stress resistance observed between termite queens and workers. Next, to demonstrate the cause of the high oxidative stress resistance of termite queens, we compared several antioxidant activities and antioxidant gene expression levels between queens and non-reproductive workers, soldiers, and nymphs. In contrast to previous reports, we were able to show that termite queens have higher antioxidant activities than non-reproductive individuals. The CAT activity of termite queens was markedly higher than in other solitary insects and the queens of eusocial Hymenoptera. We hypothesize that high activity and expression of antioxidant enzymes, especially CAT, are primed to respond rapidly and scavenge ROS that cause oxidative stress, and consequently termite queens attain both greater longevity and sustained high fecundity.

## Materials and Methods

### Sample

*Bombyx mori* (larvae, pupae and adults) and *Drosophila melanogaster* (adults; Oregon R) were provided by Prof. J Kobayashi and Prof. R Murakami, respectively. Solitary mantises *Tenodera aridifolia* (adults) were collected from grounds of Yamaguchi University. Three colonies of wasp *Vespa simillima xanthoptera* (larvae, workers, adult males, and queens) were received from an exterminator. Three colonies of ant *Camponotus obscuripes* (workers and queens) and 11 colonies of termite *Reticulitermes speratus* (workers, soldiers, nymphs, and queens [mature neotenic queens]) were collected from the experimental forest of Yamaguchi University, which is part of Mt. Himeyama in Yamaguchi, western Japan. Except as where specified in the figure legends, all insect samples were classified by sex, and one individual was used per sample, although we pooled 10 individuals of *D*. *melanogaster* adults, 5 individuals of *C*. *obscuripes* workers. In *R*. *speratus*, we used different pooled termite samples from different colonies for each experiment as described (S1 Table). For oxidative damage analysis, we prepared termite samples after 20 min irradiation with UV-B (312 nm, 10.4 kJ/ m^2^; Vilber Lourmat TF-20M) on a Petri dish. Then, to irradiate all samples equally, stimulations were performed for each group of 5 individuals of workers or a queen and we observed that individuals were alive (S1 Fig). These insect samples were preserved at –80°C until use.

### 8-Hydroxy-2’-deoxyguanosine assay

The concentration of 8-OHdG was determined in extracted insect DNA using a EpiQuik™ 8-OHdG DNA damage quantification direct kit (colorimetric) (Epigentek) in accordance with the manufacturer’s instructions. Briefly, total DNA was extracted using a DNA extractor^®^ TIS kit (Wako Pure Chemical Industries) from termite whole bodies. DNA was bound to wells that have high DNA binding affinity. Then the 8-OHdG present in the DNA was detected by using capture and detection antibodies. An enhancer solution was used to enhance the signal followed by reading the absorbance using a spectrophotometer at 450 nm within 2–15 min. The results are expressed as relative quantification (%) to the positive control provided by the kit and normalized to the input DNA (ng). Six biological replicates were performed, each with five workers and a queen (S1 Table).

### Protein carbonyl assay

Oxidative protein was quantified as PC using a protein carbonyl colorimetric assay kit (Cayman Chemical) in accordance with the manufacturer’s instructions. Briefly, termite whole bodies were homogenized in 200 μL ice-cold buffer (20 mM Tris-HCl, 1 mM EDTA, 2% protease inhibitor cocktail (v/v)). After centrifugation at 16200 *g* for 10 min at 4°C, the supernatants were placed into a new tube with 2,4-dinitrophenylhydrazine reagent followed by incubation in the dark at room temperature for 60 min. Then, 1 mL of 20% trichloroacetic acid (TCA) solution (w/v) was added to the samples before centrifugation at 16200 *g* for 10 min. The pellets were washed three times with 1 mL of (1:1) ethanol/ethyl acetate mixture. The obtained pellets were resuspended in guanidine hydrochloride solution. After vortexing and centrifugation, we measure the absorbance of the supernatant at 370 nm. The levels of PC were calculated as the amount relative to the total protein amount. Three biological replicates were performed, each with five workers and two queens (S1 Table).

### Unsaturated fatty acids quantification assay

For quantification of UFAs, we used a lipid quantification Kit (Colorimetric; Cell Biolabs) in accordance with manufacturer’s instructions. Briefly, lipid standards and lipid samples were extracted from the whole bodies of termites using 300 μL (1:1) chloroform/methanol mixture at –20°C followed by resuspension in dimethyl sulfoxide (Wako), which was incubated with 18 M sulfuric acid at 90°C for 10 min. After mixing with vanillin reagent, these samples were incubated at 37°C for 15 min. The levels of UFAs were detected at a wavelength of 540 nm and calculated from the standard curve of lipid standard. The corrected value of lipid was calculated as follows: UFA (μg)/sample weight (mg). Three biological replicates were performed, each with five workers, three nymphs, and two queens (S1 Table).

### Thiobarbituric acid reactive substances assay

As assessment of oxidative damage by lipid peroxidation was determined by using a TBARS assay kit (Cayman chemical). Briefly, termite whole bodies were homogenized in 200 μL ice-cold buffer (20 mM Tris-HCl, 2% protease inhibitor cocktail (v/v)). MDA standard or samples were mixed with 50 μL 10% SDS solution (w/v) and 1 mL color reagent (0.53% thiobarbituric acid (w/v) in 10% acetic acid solution (v/v) and 1.5% sodium hydroxide solution (v/v)), and incubated for 30 min at 100°C. Samples were incubated on ice for 10 min to stop the reaction and then centrifuged at 17000 *g* for 10 min at 25°C. The absorbance of the obtained supernatant was determined at 532 nm and levels calculated from a standard curve of the MDA standard. The corrected value of MDA was calculated as follows: MDA (nmol)/ sample weight (mg). We made three biological replications, each with five workers, three nymphs, and two queens (S1 Table).

### Protein extraction

Whole bodies of insect samples stored at –80°C were first ground to powder in liquid nitrogen and then homogenized by sonication in the tubes with buffer (20 mM Tris-HCl, 2% protease inhibitor cocktail (v/v)), followed by centrifugation at 17000 *g* for 30 min at 4°C. The supernatant containing proteins was transferred to a new tube and used as a sample. Each sample had its protein concentration measured using a BCA protein assay kit before extraction. These protein samples were preserved at –80°C until use for antioxidant activity assays.

### Antioxidant enzyme activity assays

The activities of antioxidant enzymes were determined as in a previous report [24]. Briefly, quantification of CAT activity was assayed by measuring the decomposition of hydrogen peroxide (H_2_O_2_) by monitoring absorbance at 240 nm. The reaction was started by the addition of 15 μg total protein to a reaction buffer containing 50 mM Tris-HCl (pH 7.5), 2.5 mM EDTA and 10 mM H_2_O_2_. CAT activity was defined as the rate of disappearance of H_2_O_2_ and we calculated arbitrary units relative to the value from *R*. *speratus* workers.

Prx activity was determined using an indirect assay that links Prx-mediated oxidation of thioredoxin (Trx) with the recycled reduction of Trx_ox_ (-S-S-) to Trx_red_ (-SH) by TrxR (thioredoxin reductase) using NADPH as a reductant. The absorbance at 340 nm was monitored at 30°C for 5 min. Similar to the CAT activity assay, we also calculated arbitrary units relative to the value from *R*. *speratus* workers in the Prx activity assay. Three biological replicates were performed for all insect samples classified by sex. In only queens of *R*. *speratus*, 12 and 9 replications were made for CAT and Prx activity, respectively. Except as specified in the text and figure legend, the obtained data from solitary insects and non-reproductive individuals of *R*. *speratus* classified by sex were mixed, by which the ratio of males and females was 1:1. This means that the mixed sample size becomes n = 6. We showed the distinction between males and females as supplementary information (S4 and S5 Figs).

### Quantitative real-time PCR

The whole transcriptome of *R*. *speratus* was examined using Next-generation RNA-sequencing technology in the previous study [25]. We obtained mRNA sequences of antioxidant genes from the transcriptome data through a Blast search with the amino acid sequences of translated antioxidant genes in the termite *Zootermopsis nevadensis*, and designed primer pairs for each the gene using Primer3 (version 1.1.4; [26]; S2 Table). Using ISOGEN reagent (Nippon gene), total RNA was extracted individually from whole bodies of termite workers, soldiers, nymphs, or queens which were frozen with liquid nitrogen and stored at –80°C until extraction. Immediately, cDNA was synthesized from the RNA using a PrimeScript^TM^ RT reagent kit (Takara), and preserved at –20°C. Quantitative real-time PCR (qRT-PCR) was performed using a LightCycler (Roche) with QuantiTect^®^ SYBR^®^ Green PCR (Qiagen). All procedures were performed in accordance with each manufacturer's protocol. GAPDH was selected as the reference gene. Relative expression levels were calculated using a typical ΔΔCt method. Twelve biological replicates were performed, each with three workers, three soldiers, and two nymphs of *R*. *speratus*. Nine replications were made for one queen of *R*. *speratus*. Except as specified in the text and figure legends, the obtained data from non-reproductive individuals of *R*. *speratus* classified by sex were mixed, by which the ratio of males and females was 1:1.

### Statistical analysis

R software package (version 3.2.2) was used for most statistical analyses. Unpaired t test followed by *P* value correction using Holm’s method [27] for multiple comparisons was performed on the different sets of data. All data in graphs are presented as the mean ± standard error of the mean (SEM), and all calculated *P* values are provided in figure legends. Differences were considered significance when the *P* value was * *P* < 0.05, ** *P* < 0.01.

## Results

### Oxidative DNA, protein, and lipid damage in termite queens was markedly lower than non-reproductive workers

**Fig 1:**
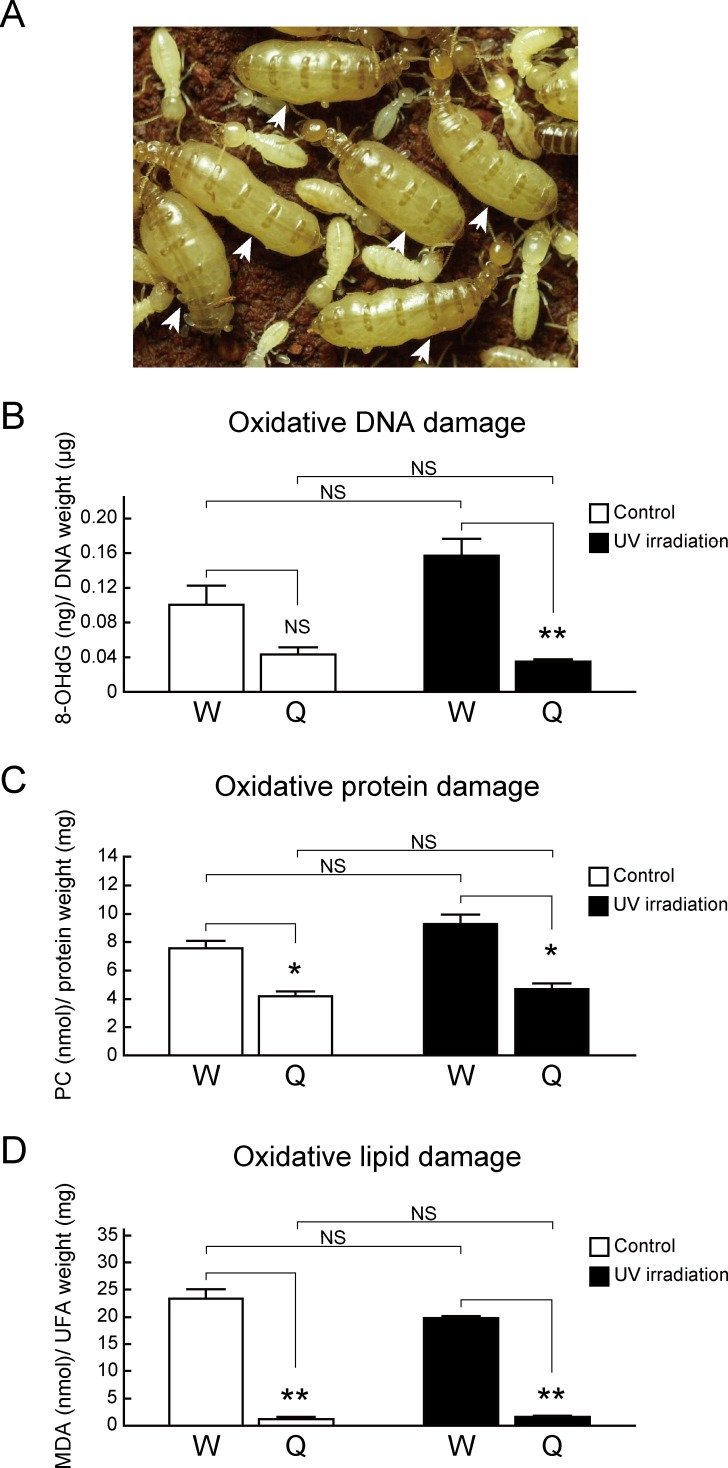
The levels of oxidative damage are different between queens and non-reproductive workers in *R*. *speratus*. Q, queens; W, workers. (A) The high caste polymorphism between queens and workers in eusocial termite *R*. *speratus*. Arrowheads indicate queens. (B) No difference in oxidative DNA damage was observed between queens and workers in control conditions (*n* = 6; for queen/worker: *P* = 0.106). However, after UV irradiation, queens showed lower levels of 8-OHdG than workers (*n* = 6; *P* < 0.001). (C) The levels of protein carboxyl were lower in the body of queens in comparison with workers in control conditions (*n* = 3; *P* = 0.019), as well as after UV irradiation (*n* = 3; *P* = 0.016). (D) Queens also had lower levels of oxidative lipid damage than workers in both control conditions (*n* = 3; *P* < 0.001) and UV irradiated conditions (*n* = 3; *P* < 0.001). We used pooled samples, shown as below (S1 Table), for each replication. White and black bars indicate control and post UV irradiation, respectively. Error bars represent standard error of the mean (SEM). Significance was measured using unpaired t test followed by Holm’s adjustment (NS, no significance; *P < 0.05, **P < 0.01).

To investigate whether high resistance to oxidative stress allows termite queens to achieve long lifespan, we performed a comparison of oxidative damage to biomolecules in *R*. *speratus* queens and workers (Fig 1A). The major biomolecules susceptible to oxidative damage are DNA [28], protein [29], and lipids [30] in most organisms. First, we assessed the levels of oxidative DNA damage using a detection assay for 8-hydroxy-2’-deoxyguanosine (8-OHdG), which is widely accepted as a sensitive marker of oxidative DNA damage. Although the 8-OHdG values in queens did not differ from the value in workers in control conditions, increased oxidative DNA damage due to UV irradiation, which produces singlet molecular oxygen and increases 8-OHdG levels [31], was suppressed only in queens but not in workers (Fig 1B). Next, we assessed the levels of oxidative protein damage by detection of protein carbonyls (PCs), which are major biomarkers of oxidative damage of protein. To measuring PCs, we performed a colorimetric assay using the reaction of 2,4-dinitrophenylhydrazine with PCs to produce hydrazone, which can be analyzed spectrophotometrically [32]. Queens showed lower levels of PCs than workers in normal conditions and post UV irradiation (Fig 1C). Lastly, we assessed the levels of oxidative lipid damage in queens and workers using a thiobarbituric acid reactive substances (TBARS) assay, which is a well-established method for screening for malondialdehyde (MDA), the end product of lipid peroxidation [33, 34]. Then, because workers showed markedly low levels of unsaturated fatty acids (UFAs) susceptible to ROS, in comparison with queens (S2 Fig), the values of MDA were revised by the UFA amounts in queens and workers. The corrected values of MDA were significantly lower in queens compared with workers (Fig 1D). Additionally, augmented MDA due to UV irradiation was suppressed in queens, whereas queens had higher and equal UFA levels in comparison with workers and nymphs, respectively (S3 Fig). Together, these results suggested that the ability to maintain lower oxidative stress in biomolecules is responsible for termite queens showing dramatically greater longevity.

### High catalase activity and *RsCAT1* gene expression levels provide an efficient antioxidant system in termite queens

**Fig 2:**
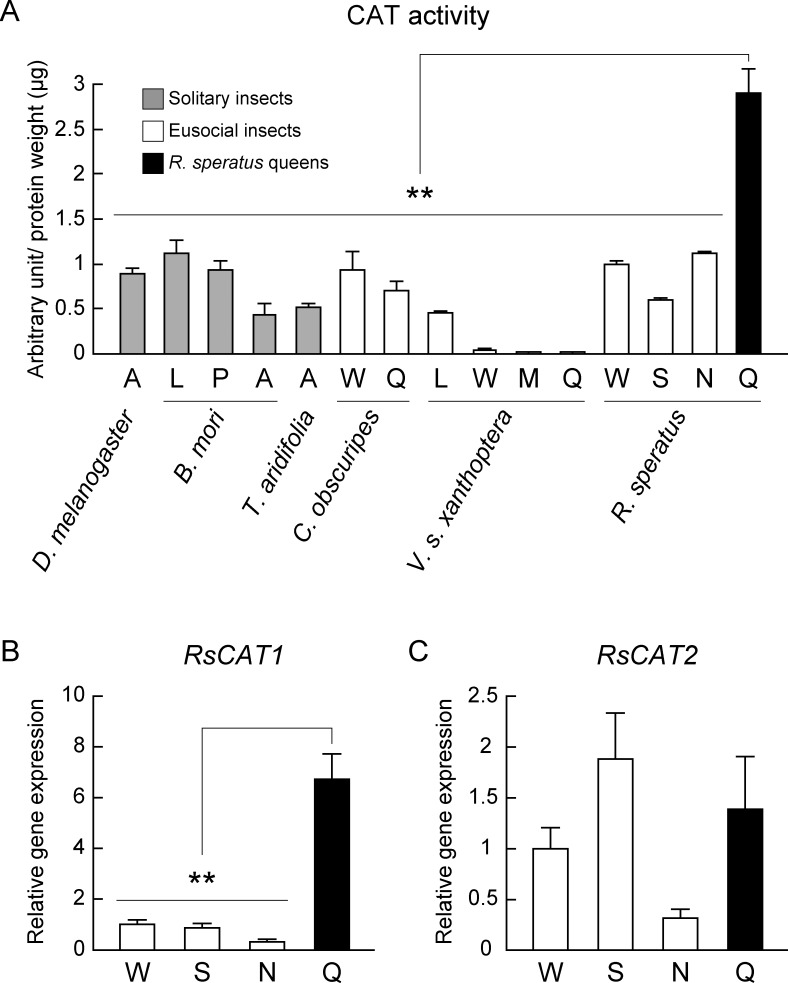
Termite queens have high CAT activity and gene *RsCAT1* expression. A, adults; L, larvae; P, pupae; W, workers; M, male adults; Q, queens; S, soldiers; N, nymphs. (A) Queens of *R*. *speratus* (*n* = 12) had markedly higher CAT activity than *D*. *melanogaster* adults (*n* = 6; 10 individuals per replicate; *P* < 0.001), *B*. *mori* larvae (*n* = 6; *P* = 0.001), *B*. *mori* pupae (*n* = 6; *P* = 0.001), *B*. *mori* adults (*n* = 6; *P* < 0.001), *T*. *aridifolia* adults (*n* = 6; *P* < 0.001), *C*. *obscuripes* workers (*n* = 6; 5 individuals per replicate; *P* = 0.001), *C*. *obscuripes* queens (*n* = 3; *P* = 0.001), *V*. *s*. *xanthoptera* larvae (*n* = 3; *P* = 0.001), *V*. *s*. *xanthoptera* workers (*n* = 3; *P* = 0.001), *V*. *s*. *xanthoptera* adult males (*n* = 3; *P* = 0.001), *V*. *s*. *xanthoptera* queens (*n* = 3; *P* = 0.001), *R*. *speratus* workers (*n* = 6; *P* = 0.001), *R*. *speratus* soldiers (*n* = 6; *P* < 0.001), and *R*. *speratus* nymphs (*n* = 6; *P* = 0.001). The values of CAT activity in solitary insects were pooled male-female data (1:1). (B) Queens of *R*. *speratus* (*n* = 9) also showed higher CAT gene *RsCAT1* expression than non-reproductive workers (*n* = 12; *P* < 0.001), soldiers (*n* = 12; *P* < 0.001), and nymphs (*n* = 12; *P* < 0.001). (C) There was no difference in CAT gene *RsCAT2* expression between queens (*n* = 9) and non-reproductive individuals (*n* = 12; for queen/worker: *P* = 0.915; for queen/soldier: *P* = 0.915; for queen/nymph: *P* = 0.092). Except as specified in the text, we used one individual of solitary insects or eusocial Hymenoptera for several replications, whereas termite samples were pooled as described below (S1 Table). All data obtained between male and female of solitary insects and non-reproductive individuals of *R*. *speratus* were mixed by which the ratio of males and females was 1:1. Gray, white, and black bars indicate solitary insects, eusocial insects, and *R*. *speratus* queens, respectively. Error bars represent standard error of the mean (SEM). Significance was measured using unpaired t test followed by Holm’s adjustment (**P < 0.01).

The major antioxidant enzymes in insects are CAT and Prx, which play a role in the management of oxidative damage [35]. Because *R*. *speratus* queens showed markedly lower levels of oxidative damage in comparison with workers (Fig 1), we next investigated whether termite queens had higher antioxidant activities than non-reproductive individuals and other insect species. Furthermore, we confirmed whether antioxidant activities were supported by gene transcription levels or not. To measure levels of antioxidant gene expression, we identified two CAT genes (*RsCAT1* and *RsCAT2*) and four Prx genes (*RsPRX1*, *RsPRX4*, *RsPRX5* and *RsPRX6*) by the method described below (S3 Table). Queens showed significantly higher CAT activity than not only termite non-reproductive individuals (workers, soldiers, and nymphs), but also other solitary insects (*Drosophila melanogaster*, *Bombyx mori*, and *Tenodera aridifolia*) and eusocial queens of Hymenoptera (*Vespa simillima xanthoptera* and *Camponotus obscuripes*) (Fig 2A). Then, the values of CAT activity in solitary insects were pooled for male-female data (1:1). Separate male and female data for CAT activity are shown in S4 Fig. These results indicated that a different antioxidant system to protect biomolecules from oxidative stress has evolved between eusocial Isoptera and Hymenoptera. Next, we investigated CAT gene expression levels between individuals of *R*. *speratus*. As a result, we found that queens had a significantly higher level of *RsCAT1* expression but not *RsCAT2* expression, which was not consistent with their CAT activity (Fig 2B and 2C). A previous report demonstrated that CAT activity is essential for longevity and fertility in sand fly [36, 37], suggest the possibility that the high CAT activity and *RsCAT1* expression are important for termite queens for both an extraordinary long lifespan and high fertility.

### Termite queens have high Prx gene *RsPRX6* expression in comparison with non-reproductive individuals

**Fig 3:**
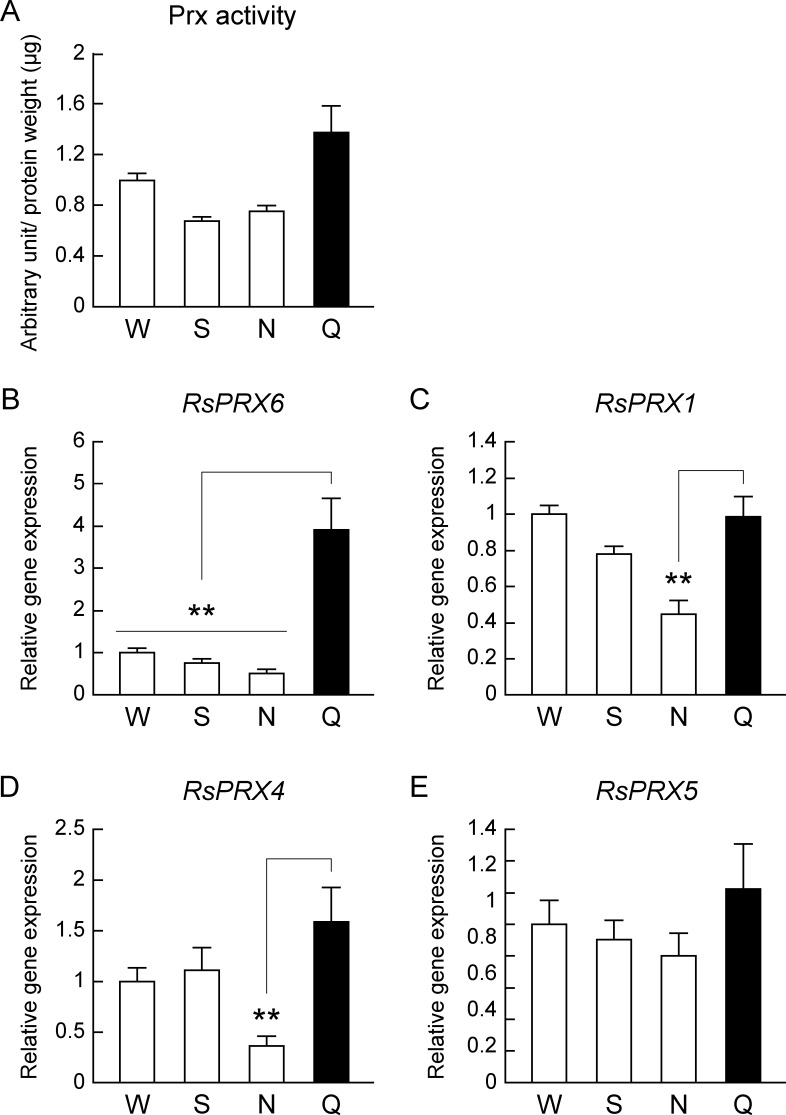
Termite queens have high level of 1-Cys Prx gene *RsPRX6* expression. W, workers; S, soldiers; N, nymphs; Q, queens. (A) There was no difference in Prx activity between queens (*n* = 9) and non-reproductive individuals (*n* = 3; for queen/worker: *P* = 0.342; for queen/soldier: *P* = 0.279; for queen/nymph: *P* = 0.279). (B) Queens (*n* = 9) had higher levels of *RsPRX6* gene expression than workers (*n* = 12; *P* < 0.001), soldiers (*n* = 12; *P* < 0.001), and nymphs (*n* = 12; *P* < 0.001). (C) Queens (*n* = 9) also had higher *RsPRX1* gene expression than nymphs (*n* = 12; *P* = 0.002) but not workers (*n* = 12; *P* = 0.885) or soldiers (*n* = 12; *P* = 0.149). (D) The level of *RsPRX4* gene expression in queens (*n* = 9) was also higher than nymphs (*n* = 12; *P* = 0.003) but not workers (*n* = 12; *P* = 0.184) or soldiers (*n* = 12; *P* = 0.236). (E) There was no difference in *RsPRX5* gene expression between queens (*n* = 9) and non-reproductive individuals (*n* = 12; for queen/worker: *P* = 0.555; for queen/soldier: *P* = 0.555; for queen/nymph: *P* = 0.524). We used pooled samples for each replication, shown as below (S1 Table), for several replications. All data obtained between male and female of non-reproductive individuals were mixed by which the ratio of males and females was 1:1. White and black bars indicate non-reproductive individuals and queens, respectively. Error bars represent standard error of the mean (SEM). Significance was measured by unpaired t test followed by Holm’s adjustment (**P < 0.01)

As a result of a continuous study of antioxidant enzymes, we investigated Prx activity in *R*. *speratus*. Here, we found that Prx activity of *R*. *speratus* queens was slightly higher, but not significantly different, than in non-reproductive individuals (Fig 3A). There was also no difference in the comparative analysis between insect species (S5 Fig). Of note, *R*. *speratus* queens showed markedly higher expression levels of the Prx gene *RsPRX6*, which belongs to the 1-Cys Prx subgroup and has been reported as a factor that rescues declining brain function with advancing age in honeybees [38] (Fig 3B). *R*. *speratus* queens showed higher expression levels of *RsPRX1* and *RsPRX4*, which belong to the typical 2-Cys Prx subgroups, than in nymphs but not workers and soldiers (Fig 3C and 3D). There was no difference of the expression level of *RsPRX5* belonging to the atypical 2-Cys Prx subgroup between queens and non-reproductive individuals (Fig 3E).

A previous report described GPx activity as almost absent in insects [39]. However, we investigated the expression level of two GPx genes, *RsGPX* and *RsPHGPX*, to confirm if this is also the case in termite individuals. Non-reproductive individuals had higher levels of *RsGPX* and *RsPHGPX* gene expression than queens (S6 Fig). GPx activity is too low to be measured in invertebrates [39]; therefore, we considered that the difference in the expression levels among *R*. *speratus* castes was not important for their antioxidant system.

Taken together, these findings suggested that higher *RsPRX6* gene expression plays an important role in the efficient antioxidant system of termite queens in order to attain great longevity despite their fertile phenotype, as well as CAT activity and *RsCAT1* expression.

## Discussion

Although the question of how social insect queens achieve long lifespan in comparison with non-reproductive individuals has attracted much attention, the molecular mechanisms involved are not yet understood. Recently, several studies about this mechanism have been reported using ants [2, 22, 40] and honeybees [23, 41–43]. Nevertheless, to our knowledge, no research has been published on termite queens, which exhibit extraordinary longevity and fertility as well as ants and honeybees. In the present study, we demonstrated for the first time that an efficient antioxidant system may partly explain this phenomenon in the eusocial subterranean termite *R*. *speratus*. The oxidative stress theory is a major aging hypothesis and suggests that an efficient antioxidant system contributes to lifespan extension in many organisms including insects [35]. Generally, oxidative stress is caused by over-generation of ROS, and the accumulation of oxidative damage to biomolecules is associated with aging and longevity [12–15]. Here, we revealed that the termite queens maintain lower levels of 8-OHdG than workers after UV irradiation and also constantly maintain lower levels of PC and MDA (Fig 1).

From this result, we hypothesized that the queens have a highly efficient antioxidant system. Therefore, we paid attention to the antioxidant enzymes CAT and Prx, which are thought to be major components of the antioxidant system in insects [35], and we investigated whether queens have high antioxidant enzyme activity. We demonstrated that queens had higher CAT activity and *RsCAT1* gene expression levels than non-reproductive individuals in *R*. *speratus* (Fig 2A and 2B). Surprisingly, CAT activity of queens was also markedly higher in comparison with other solitary insects and eusocial Hymenoptera (Fig 2A). These results indicated that CAT plays a role in the efficient antioxidant system in *R*. *speratus*. A previous study reported that CAT plays a central role in protecting the oocyte and early embryo from ROS damage in the mosquito *Anopheles gambiae* [37]. Furthermore, another study proposed that CAT is important for female fecundity and mortality in the phlebotomine sand fly *Lutzomyia longipalpis* [36]. These studies also supported our hypothesis that termite queens have an efficient antioxidant system to attain greater longevity.

Although Prx activity in queens was non-significantly higher than the activities in non-reproductive individuals, queens had higher expression levels of *RsPRX6* encoding 1Cys-Prx (Fig 3A and 3B). Abundant 1Cys-Prx expression during embryogenesis was reported in *D*. *melanogaster* [44], suggesting that *RsPRX6* may be associated with a high rate of cell proliferation during embryogenesis, consistent with the fertile phenotype of termite queens. The levels of 1Cys-Prx expression rescue declining brain function at advanced age in honeybees [38], which also supports the anti-aging phenotype of termite queens. Consequently, these results propose that, because termite queens have an efficient antioxidant system composed of antioxidant enzymes, especially CAT, queens achieve striking longevity.

Previously, Parker et al. showed that long-lived queens do not have higher Cu/Zn-SOD activity and SOD gene expression than short-lived adult workers and males in the black garden ant *Lasius niger* [22]. Corona et al. also obtained similar results in the honeybee *Apis mellifera* [23]. These two reports indicated that the antioxidant enzymes are not relevant to the unusual characteristics of social insect queens. These findings, which are in contradiction with our results, suggested the possibility that the antioxidant systems of termites are partially different from the antioxidant system of ants and honeybees (or wasps). On the other hand, because we investigated only CAT and Prx in *R*. *speratus* in this present study, further studies are needed to evaluate other antioxidants such as SOD and vitellogenin, which is a precursor of yolk protein that is thought to be important for social evolution in all social insects [45].

We observed similar antioxidant activity in termite queens compared with other insects (S5 Fig). Aerobic respiration is one of the major sources of ROS resulting in oxidative stress [46] and it also has an important role in an organism’s lifespan [13]. It is unclear why termite queens do not have higher antioxidant activities than other insect species, but one possibility is that termites, which generally live in hypoxic subterranean habitats (e.g., in wood), might repress their aerobic respiration causing ROS production. Interestingly, several termite species indicated ubiquitously higher respiratory quotients (the rate between oxygen consumption and carbon dioxide emission) above 1.00 [47], suggesting that termites may be capable of repressing aerobic respiration. For this reason, we expected slightly lower levels of ROS generation in the termite body. Therefore, further studies are needed to evaluate the level of ROS production between short-lived and long-lived insects. Moreover, because lower termites such as *R*. *speratus* have a lot of gut symbionts [48], it remains to be determined whether the antioxidant ability of termites depends on their gut symbiont. Furthermore, we used termite samples that were age-indeterminate in this present study. Thus, long-term studies are also needed to determine the true longevity of termite reproductives in the future.

This comparative study exploits the untapped resource of natural variation in longevity in the eusocial termite *R*. *speratus*. To the best of our knowledge, we have revealed for the first time that termite queens suffer lower levels of oxidative damage than non-reproductive workers, and that an efficient antioxidant system consisting of several antioxidant enzymes, especially high CAT activity from *RsCAT1* gene expression, may play an important role in their oxidative stress resistance. These findings highlight not only the question of how termite queens achieve long lifespan, but also the evolutionary linkage of reproductive division of labor in social insects.

## Supporting Information

S1 Fig Survival rate after UV irradiation in *R*. *speratus* workers. Average survival was calculated immediately after 0, 5, 10, 15, 20, 15, and 30 min UV-B irradiation (312 nm, 10.4 kJ/ m^2^; Vilber Lourmat TF-20M). Although we observed 100%, 98%, and 93% survival of workers after 0–15, 20, and 25 min irradiation, respectively, workers irradiated for 30 min showed only 43% survival (*P* < 0.001). Six biological replicates were performed for each group of 10 individuals of workers on a Petri dish. Error bars represent standard error of the mean (SEM). Significance was measured by unpaired t test (NS, no significance; **P < 0.01).
(TIF)

S2 Fig Results of unsaturated fatty acids quantification assay. Queens had higher levels of UFAs susceptible to oxidation than non-reproductive workers (*P* = 0.003) but not nymphs (*P* = 0.719). These data suggested why irradiation cannot increase the malondialdehyde (MDA) levels in workers (S3 Fig). We used pooled samples, shown as below (S1 Table), for 3 replications. Error bars represent standard error of the mean (SEM). Significance was measured by unpaired t test followed by Holm’s adjustment (NS, no significance; **P < 0.01).
(TIF)

S3 Fig Results of Thiobarbituric acid reactive substances (TBARS) assay. TBARS assays demonstrated that queens had lower levels of malondialdehyde (MDA) than workers (*P* = 0.002) and nymphs (*P* = 0.002) in control conditions. Moreover, after UV irradiation, we found that queens also had a potential to maintain lower MDA levels than workers (*P* < 0.001) and nymphs (*P* = 0.005). We used pooled samples, shown as below (S1 Table), for 3 replications. W, workers; N, nymphs; Q, queens. White and black bars indicate control and post UV irradiation, respectively. Error bars represent standard error of the mean (SEM). Significance was measured by unpaired t test followed by Holm’s adjustment (**P < 0.01).
(TIF)

S4 Fig Measurement of CAT activity in several insects. Termite queens (*n* = 12) had higher CAT activity than *D*. *melanogaster* adult males (*n* = 3; *P* = 0.013), *D*. *melanogaster* adult females (*n* = 3; *P* = 0.011), *B*. *mori* larvae males (*n* = 3; *P* = 0.016), *B*. *mori* larvae females (*n* = 3; *P* = 0.011), *B*. *mori* pupae males (*n* = 3; *P* = 0.014), *B*. *mori* pupae females (*n* = 3; *P* = 0.011), *B*. *mori* adult males (*n* = 3; *P* = 0.011), *B*. *mori* adult females (*n* = 3; *P* = 0.003), *T*. *aridifolia* adult males (*n* = 3; *P* = 0.008), *T*. *aridifolia* adult females (*n* = 3; *P* = 0.006), *C*. *obscuripes* workers (*n* = 6; *P* = 0.002), *C*. *obscuripes* queens (*n* = 3; *P* = 0.0011), *V*. *s*. *xanthoptera* larvae (*n* = 3; *P* = 0.006), *V*. *s*. *xanthoptera* workers (*n* = 3; *P* = 0.002), *V*. *s*. *xanthoptera* adult males (*n* = 3; *P* = 0.002), and *V*. *s*. *xanthoptera* queens (*n* = 3; *P* = 0.002). Black, white, and gray bars indicate male, female, and unknown-sex, respectively. Error bars represent standard error of the mean (SEM). Significance was measured by unpaired t test followed by Holm’s adjustment (*P < 0.05, **P < 0.01).
(TIF)

S5 Fig Measurement of Prx activity in several insects. There is no difference in Prx activity between termite queens and other insects. Termite queens had almost the same activity as *D*. *melanogaster* adult males (*n* = 3; *P* = 0.396), *D*. *melanogaster* adult females (*n* = 3; *P* = 0.359), *B*. *mori* larvae males (*n* = 3; *P* = 0.267), *B*. *mori* larvae females (*n* = 3; *P* = 1.000), *B*. *mori* pupae males (*n* = 3; *P* = 0.169), *B*. *mori* pupae females (*n* = 3; *P* = 1.000), *B*. *mori* adult males (*n* = 3; *P* = 0.879), *B*. *mori* adult females (*n* = 3; *P* = 0.403), *T*. *aridifolia* adult males (*n* = 3; *P* = 0.179), *T*. *aridifolia* adult females (*n* = 3; *P* = 0.793), *C*. *obscuripes* workers (*n* = 3; *P* = 1.000), *C*. *obscuripes* queens (*n* = 3; *P* = 1.000), *V*. *s*. *xanthoptera* larvae (*n* = 3; *P* = 1.000), *V*. *s*. *xanthoptera* workers (*n* = 3; *P* = 0.359), *V*. *s*. *xanthoptera* adult males (*n* = 3; *P* = 1.000), and *V*. *s*. *xanthoptera* queens (*n* = 3; *P* = 1.000). Black, white, and gray bars indicate male, female, and unknown-sex, respectively. Error bars represent standard error of the mean (SEM). Significance was measured by unpaired t test followed by Holm’s adjustment (*P < 0.05, **P < 0.01).
(TIF)

S6 Fig The level of GPX genes *RsGPX* and *RsPHGPX* expression in *R*. *speratus*. The levels of GPx gene expression were equal or lower in queens compared with non-reproductive individuals. W, workers; S, soldiers; N, nymphs; Q, queens (A) Queens (*n* = 6) had no significant difference in the levels of *RsGPX* expression in comparison with soldiers (*n* = 12; *P* = 0.068) and nymphs (*n* = 12; *P* = 0.103). Nevertheless, queens showed lower levels than workers (*n* = 12; *P* = 0.039). (B) Queens (*n* = 6) had almost the same levels of *RsPHGPX* expression than nymphs (*n* = 12; *P* = 0.138). However, queens had slightly lower levels of *RsPHGPX* expression than workers (*n* = 12; *P* = 0.040) and soldiers (*n* = 12; *P* = 0.049). We used pooled samples, shown as below (S2 Table), for several replications. All data obtained between male and female of non-reproductive individuals were mixed by which the ratio of males and females was 1:1. White and black bars indicate non-reproductive individuals and queens, respectively. Error bars represent standard error of the mean (SEM). Significance was measured by unpaired t test followed by Holm’s adjustment (*P < 0.05).
(TIF)

S1 Table Termite sample list. (DOCX)

S2 Table Sequences of primers used in this study. (DOCX)

S3 Table Target gene information for this study. (DOCX)
