# Supplementary figures and images for: An Efficient Antioxidant System in a Long-Lived Termite Queen
Source: PLoS One. 2017 Jan 11;12(1):e0167412. doi: 10.1371/journal.pone.0167412 (PMC5226355; doi:10.1371/journal.pone.0167412)

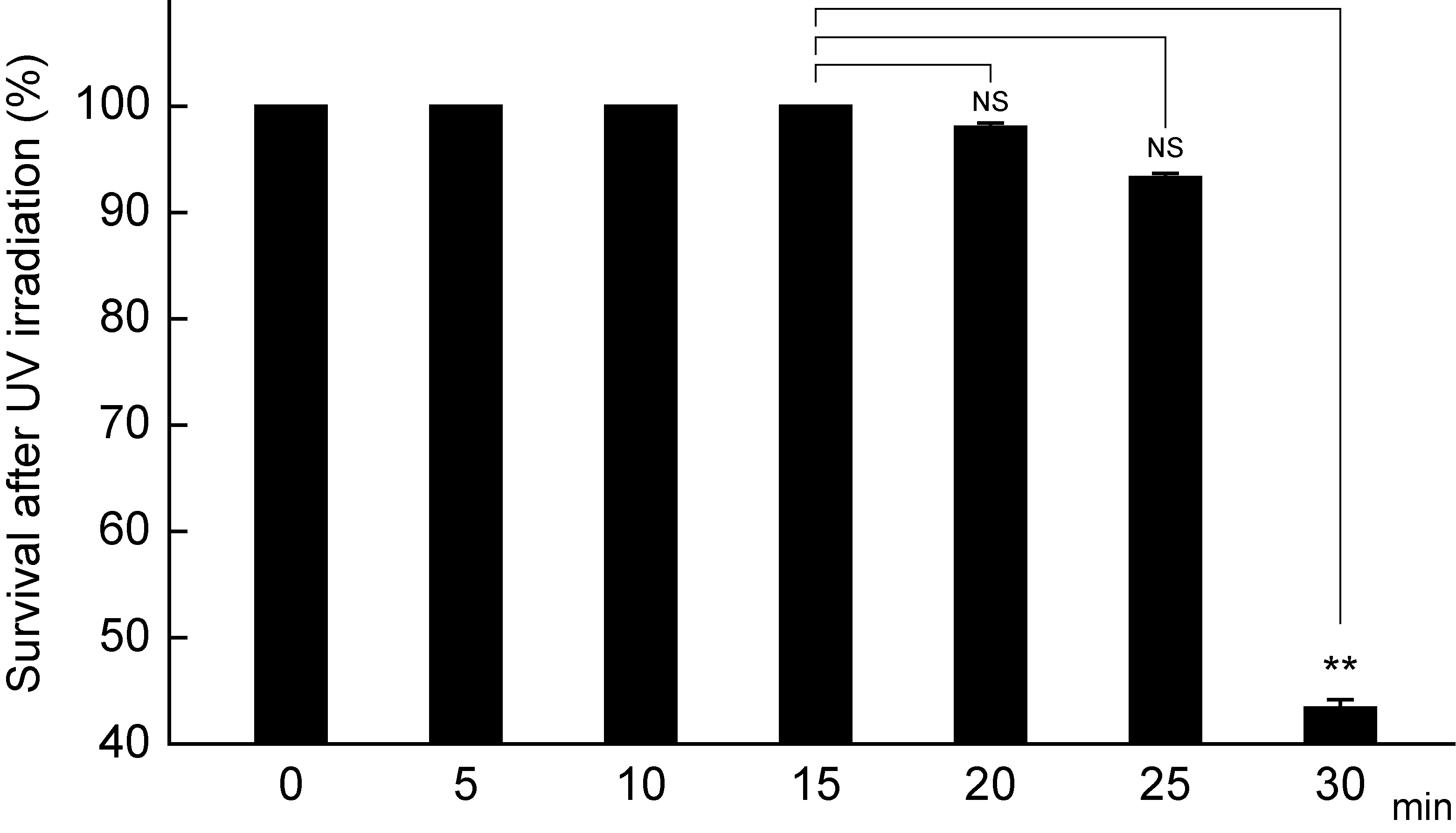

Supplement: S1 Fig — Average survival was calculated immediately after 0, 5, 10, 15, 20, 15, and 30 min UV-B irradiation (312 nm, 10.4 kJ/ m2; Vilber Lourmat TF-20M). Although we observed 100%, 98%, and 93% survival of workers after 0–15, 20, and 25 min irradiation, respectively, workers irradiated for 30 min showed only 43% survival (P < 0.001). Six biological replicates were performed for each group of 10 individuals of workers on a Petri dish. Error bars represent standard error of the mean (SEM). Significance was measured by unpaired t test (NS, no significance; **P < 0.01). (TIF) [file pone.0167412.s001.tif]

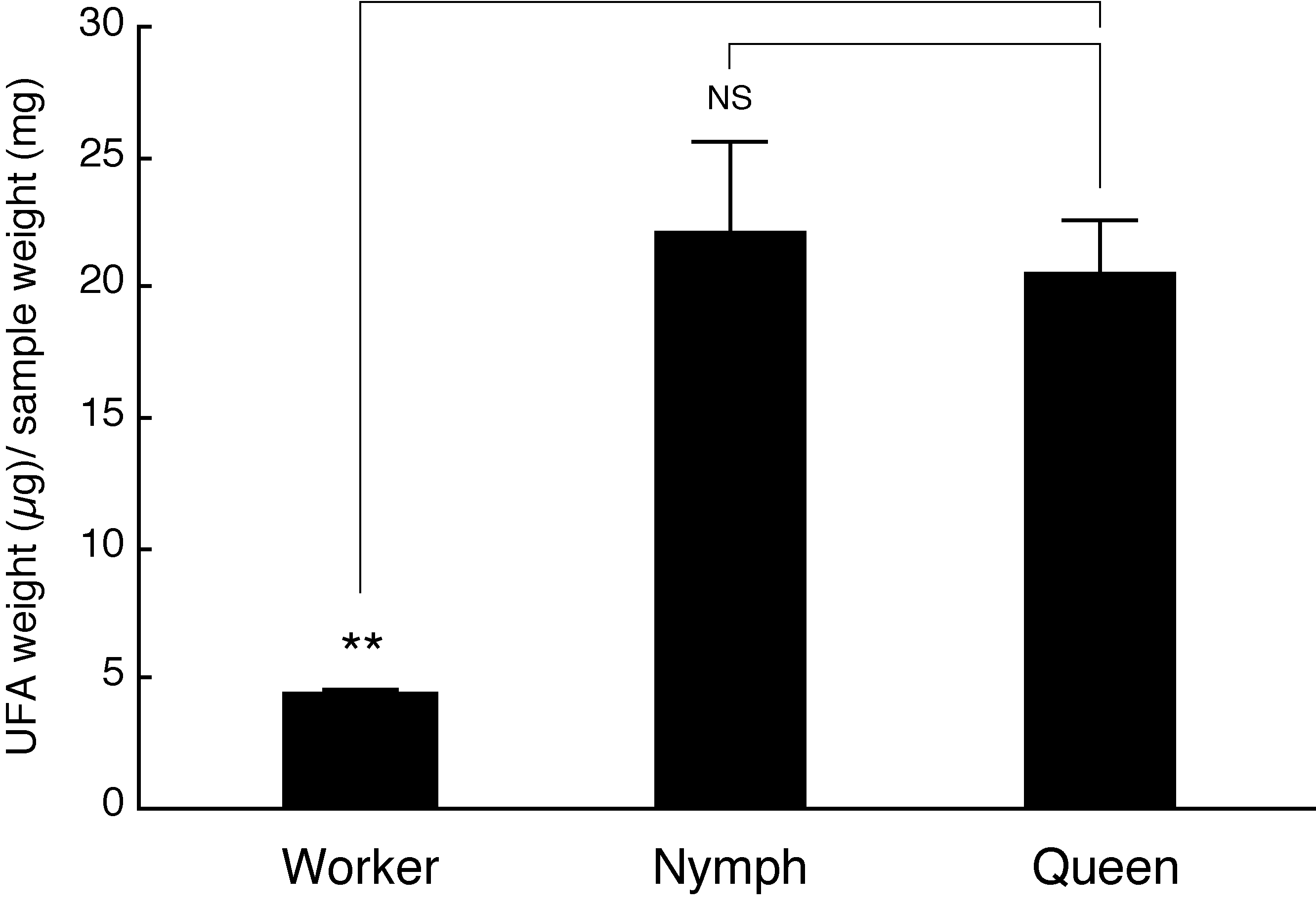

Supplement: S2 Fig — Queens had higher levels of UFAs susceptible to oxidation than non-reproductive workers (P = 0.003) but not nymphs (P = 0.719). These data suggested why irradiation cannot increase the malondialdehyde (MDA) levels in workers (S3 Fig). We used pooled samples, shown as below (S1 Table), for 3 replications. Error bars represent standard error of the mean (SEM). Significance was measured by unpaired t test followed by Holm’s adjustment (NS, no significance; **P < 0.01). (TIF) [file pone.0167412.s002.tif]

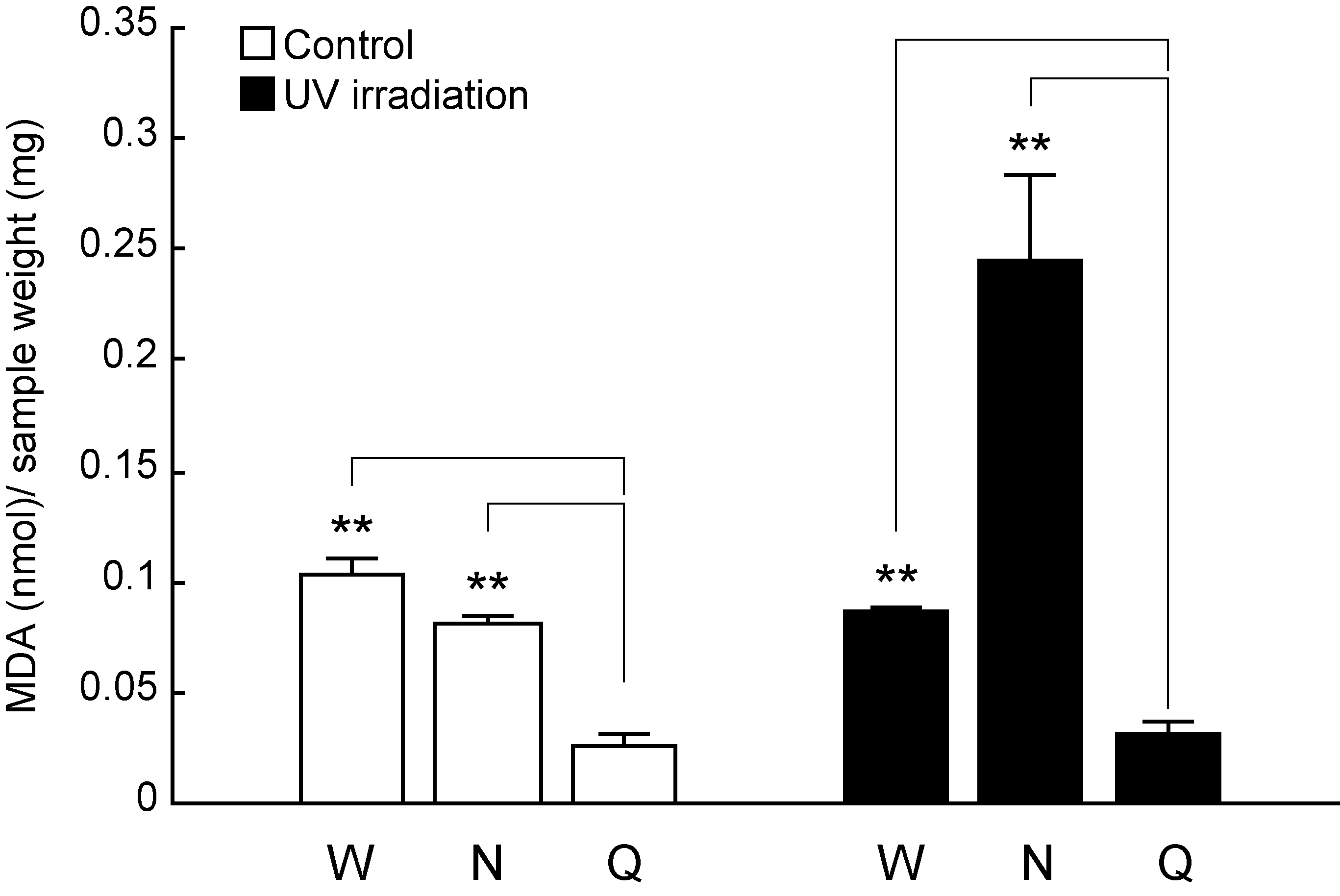

Supplement: S3 Fig — TBARS assays demonstrated that queens had lower levels of malondialdehyde (MDA) than workers (P = 0.002) and nymphs (P = 0.002) in control conditions. Moreover, after UV irradiation, we found that queens also had a potential to maintain lower MDA levels than workers (P < 0.001) and nymphs (P = 0.005). We used pooled samples, shown as below (S1 Table), for 3 replications. W, workers; N, nymphs; Q, queens. White and black bars indicate control and post UV irradiation, respectively. Error bars represent standard error of the mean (SEM). Significance was measured by unpaired t test followed by Holm’s adjustment (**P < 0.01). (TIF) [file pone.0167412.s003.tif]

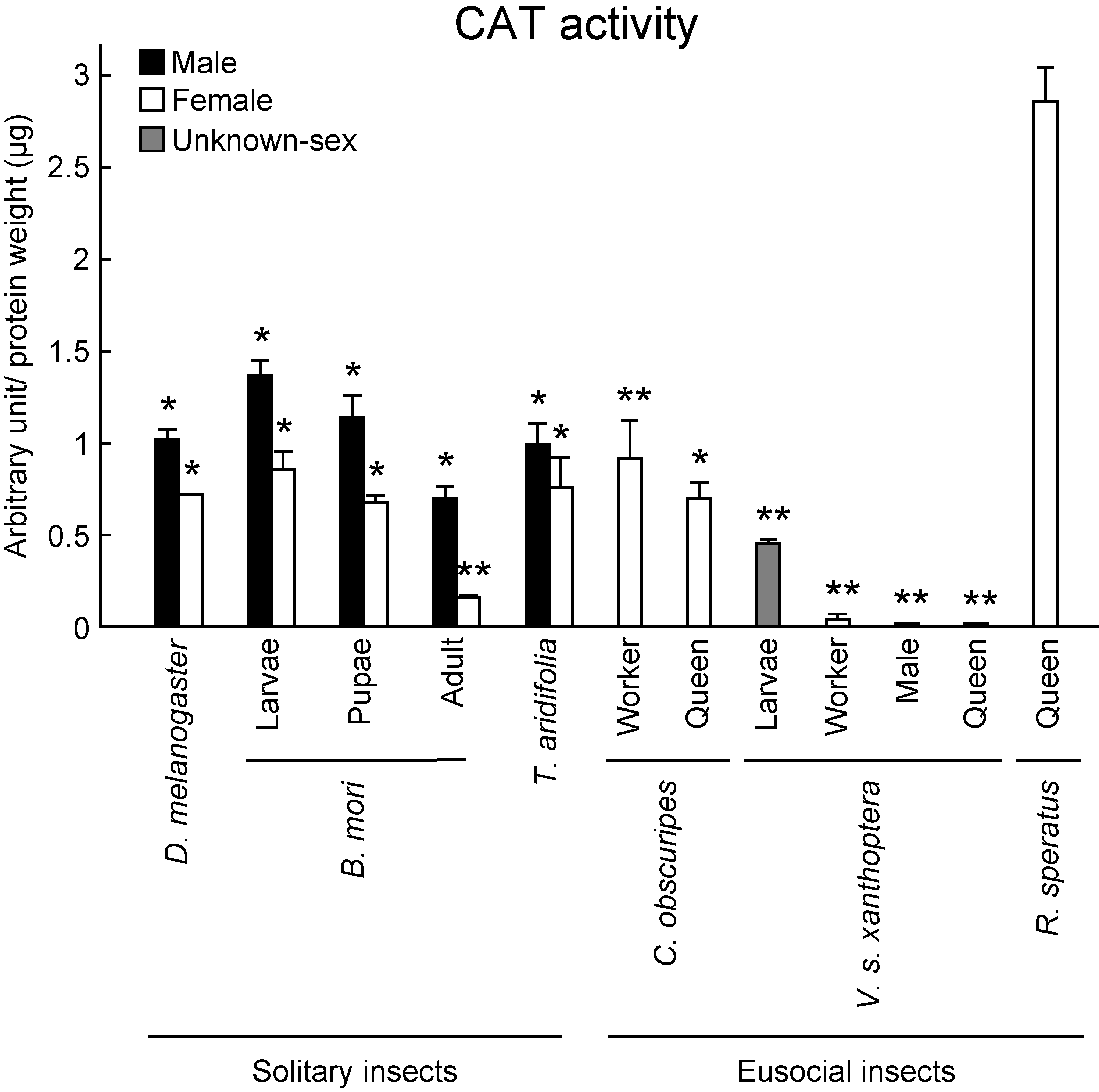

Supplement: S4 Fig — Termite queens (n = 12) had higher CAT activity than D. melanogaster adult males (n = 3; P = 0.013), D. melanogaster adult females (n = 3; P = 0.011), B. mori larvae males (n = 3; P = 0.016), B. mori larvae females (n = 3; P = 0.011), B. mori pupae males (n = 3; P = 0.014), B. mori pupae females (n = 3; P = 0.011), B. mori adult males (n = 3; P = 0.011), B. mori adult females (n = 3; P = 0.003), T. aridifolia adult males (n = 3; P = 0.008), T. aridifolia adult females (n = 3; P = 0.006), C. obscuripes workers (n = 6; P = 0.002), C. obscuripes queens (n = 3; P = 0.0011), V. s. xanthoptera larvae (n = 3; P = 0.006), V. s. xanthoptera workers (n = 3; P = 0.002), V. s. xanthoptera adult males (n = 3; P = 0.002), and V. s. xanthoptera queens (n = 3; P = 0.002). Black, white, and gray bars indicate male, female, and unknown-sex, respectively. Error bars represent standard error of the mean (SEM). Significance was measured by unpaired t test followed by Holm’s adjustment (*P < 0.05, **P < 0.01). (TIF) [file pone.0167412.s004.tif]

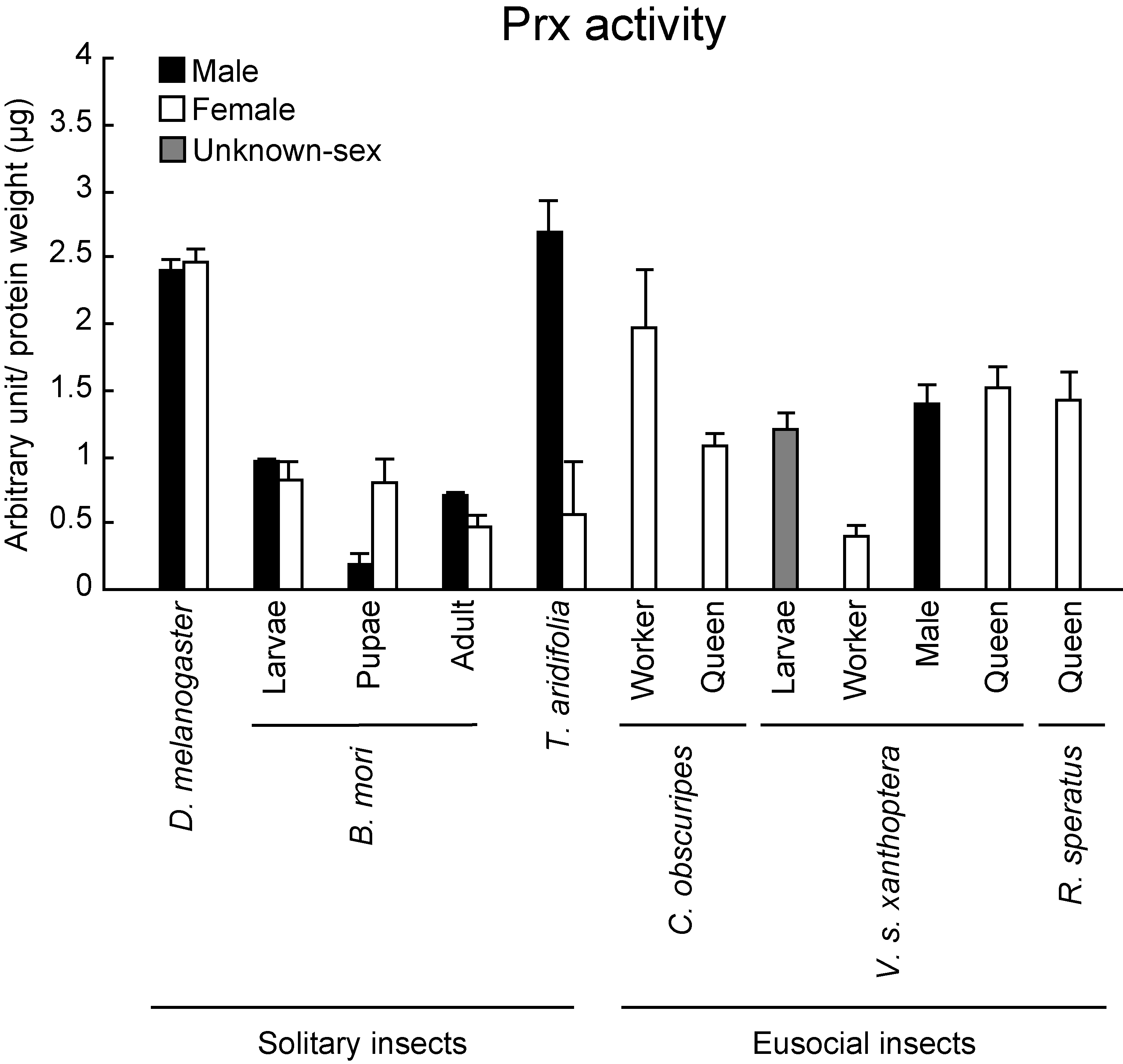

Supplement: S5 Fig — There is no difference in Prx activity between termite queens and other insects. Termite queens had almost the same activity as D. melanogaster adult males (n = 3; P = 0.396), D. melanogaster adult females (n = 3; P = 0.359), B. mori larvae males (n = 3; P = 0.267), B. mori larvae females (n = 3; P = 1.000), B. mori pupae males (n = 3; P = 0.169), B. mori pupae females (n = 3; P = 1.000), B. mori adult males (n = 3; P = 0.879), B. mori adult females (n = 3; P = 0.403), T. aridifolia adult males (n = 3; P = 0.179), T. aridifolia adult females (n = 3; P = 0.793), C. obscuripes workers (n = 3; P = 1.000), C. obscuripes queens (n = 3; P = 1.000), V. s. xanthoptera larvae (n = 3; P = 1.000), V. s. xanthoptera workers (n = 3; P = 0.359), V. s. xanthoptera adult males (n = 3; P = 1.000), and V. s. xanthoptera queens (n = 3; P = 1.000). Black, white, and gray bars indicate male, female, and unknown-sex, respectively. Error bars represent standard error of the mean (SEM). Significance was measured by unpaired t test followed by Holm’s adjustment (*P < 0.05, **P < 0.01). (TIF) [file pone.0167412.s005.tif]

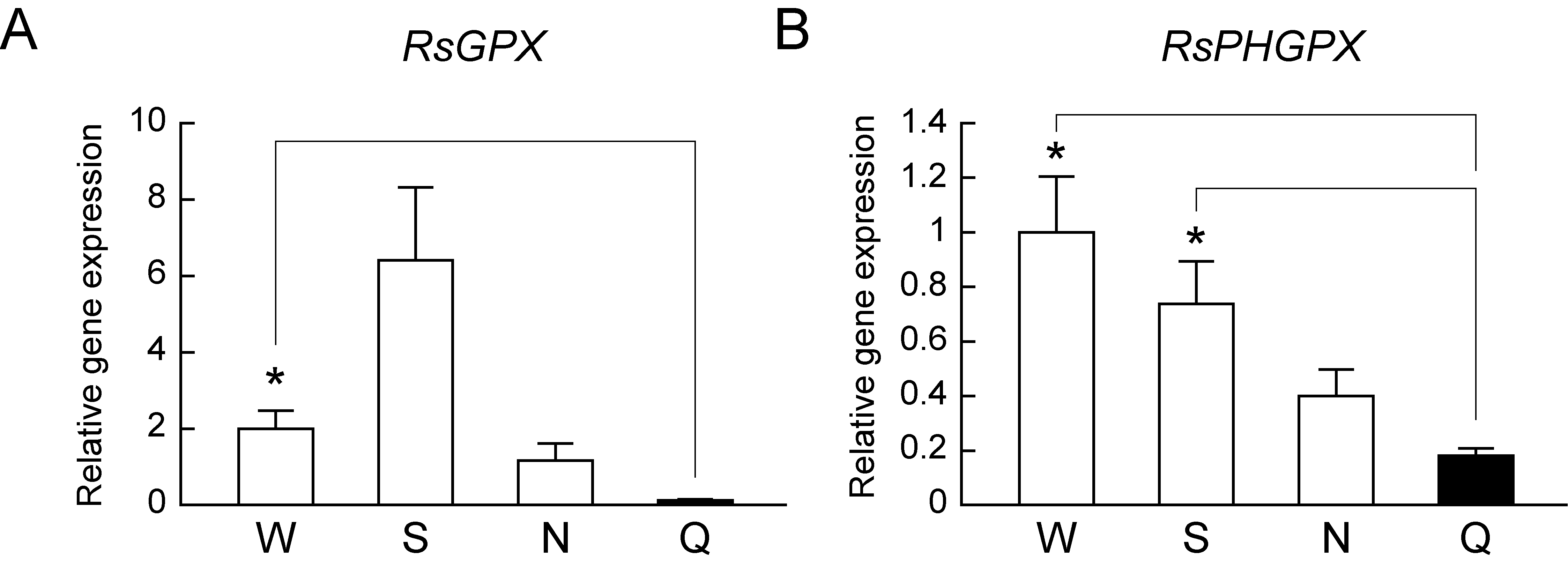

Supplement: S6 Fig — The levels of GPx gene expression were equal or lower in queens compared with non-reproductive individuals. W, workers; S, soldiers; N, nymphs; Q, queens (A) Queens (n = 6) had no significant difference in the levels of RsGPX expression in comparison with soldiers (n = 12; P = 0.068) and nymphs (n = 12; P = 0.103). Nevertheless, queens showed lower levels than workers (n = 12; P = 0.039). (B) Queens (n = 6) had almost the same levels of RsPHGPX expression than nymphs (n = 12; P = 0.138). However, queens had slightly lower levels of RsPHGPX expression than workers (n = 12; P = 0.040) and soldiers (n = 12; P = 0.049). We used pooled samples, shown as below (S2 Table), for several replications. All data obtained between male and female of non-reproductive individuals were mixed by which the ratio of males and females was 1:1. White and black bars indicate non-reproductive individuals and queens, respectively. Error bars represent standard error of the mean (SEM). Significance was measured by unpaired t test followed by Holm’s adjustment (*P < 0.05). (TIF) [file pone.0167412.s006.tif]
