# Supplementary material for: An Efficient Antioxidant System in a Long-Lived Termite Queen
Source: PLoS One. 2017 Jan 11;12(1):e0167412. doi: 10.1371/journal.pone.0167412 (PMC5226355; doi:10.1371/journal.pone.0167412)
Supplement: S1 Table — (DOCX) [file pone.0167412.s007.docx]

**S1 Table. Termite sample list**

| Analysis | Colony ID | Termite samples (pooled number of individuals per replications) |
| --- | --- | --- |
| TBARS assay and lipid quantification | YY141021A | Worker (5), nymph (3), and queen (2) |
| Protein carbonyl assay | YY150910A | Worker (5) and queen (2) |
| 8-OHdG assay | YY151022A | Worker (5) and queen (1) |
| 8-OHdG assay | YY160729A | Worker (5) and queen (1) |
| CAT and Prx activities | YY140731A | Worker (10), soldier (8), nymph (5), and queen (4) |
| CAT and Prx activities | YY140914A | Queen (4) |
| CAT and Prx activities | YY141014A | Queen (4) |
| CAT activity | YY140930A | Queen (4) |
| CAT and Prx genes expression | YY130807A | Worker (3), soldier (3), nymph (2), and queen (1) |
| CAT and Prx genes expression | YY151118A | Worker (3), soldier (3), nymph (2), and queen (1) |
| CAT and Prx genes expression | YY140919A | Queen (1) |

Numbers in Colony IDs indicate the dates when the colonies were collected (e.g., colony YY141021A was collected on October 21, 2014).
