# Supplementary material for: An Efficient Antioxidant System in a Long-Lived Termite Queen
Source: PLoS One. 2017 Jan 11;12(1):e0167412. doi: 10.1371/journal.pone.0167412 (PMC5226355; doi:10.1371/journal.pone.0167412)
Supplement: S2 Table — (DOCX) [file pone.0167412.s008.docx]

**S2 Table. Sequences of primers used in this study**

| Target gene | Sequence (5’–3’) | Amplicon (bp) |
| --- | --- | --- |
| *RsCAT1* | Forward; TGGCTATGGCTCTCACACATTC | 91 |
|  | Reverse; TTTGATGCCTTGGTCTGTCTTG |  |
| *RsCAT2* | Forward; CGGGGCTTTGCTGTGAA | 84 |
|  | Reverse; GTCCCTGATAAAGAAGATTGGTGTG |  |
| *RsPRX1* | Forward; TCTGACCGTGTAGCCGAGTTT | 106 |
|  | Reverse; CCTTCTTGCGTGGAGTGTTG |  |
| *RsPRX4* | Forward; TGTATGCCCTACAGAAATTCTTGC | 131 |
|  | Reverse; CCTTCCTTGGTGTGTTTGTCC |  |
| *RsPRX5* | Forward; GCTGTAAGGCGTGTAGTGTTGTCT | 85 |
|  | Reverse; GCTTGTCACCAACCTGAATAACC |  |
| *RsPRX6* | Forward; GAATTCCCGAATTTCAAAGCAG | 97 |
|  | Reverse; CCGAAGGATGCGAAAACAA |  |
| *RsGPX* | Forward; ATGTTCGGCCAGGGAATG | 117 |
|  | Reverse; TTGTGGAAGGGCAATGTTTCT |  |
| *RsPHGPX* | Forward; GAAATAGTGTGCTTTGCCAGGTC | 120 |
|  | Reverse; AGTTCCTCCTTGCTTGTGCTTC |  |
| *RsGAPDH* | Forward; CCATAGAAAAGGCTTCTGCACATT | 89 |
|  | Reverse; AACAACAAACATTGGGGCATC |  |
