# Supplementary material for: An Efficient Antioxidant System in a Long-Lived Termite Queen
Source: PLoS One. 2017 Jan 11;12(1):e0167412. doi: 10.1371/journal.pone.0167412 (PMC5226355; doi:10.1371/journal.pone.0167412)
Supplement: S3 Table — (DOCX) [file pone.0167412.s009.docx]

**S3 Table. Target gene information for this study**

| Target gene | Target gene ID | Accession no. | Query genes (GeneBank no.) | Query species |
| --- | --- | --- | --- | --- |
| *RsCAT1* | comp783914_c0_seq4 | FX983162 | Catalase (KDR07976.1) | *Z. nevadensis* |
| *RsCAT2* | comp808329_c0_seq1 | FX983163 | Catalase (KDR21530.1) | *Z. nevadensis* |
| *RsPRX1* | comp811232_c3_seq2 | FX983166 | Typical 2Cys-Peroxiredoxin (KDR08683.1) | *Z. nevadensis* |
| *RsPRX4* | comp795973_c0_seq1 | FX983167 | Typical 2Cys-Peroxiredoxin (KDR20462.1) | *Z. nevadensis* |
| *RsPRX5* | comp804180_c8_seq4 | FX983168 | Atypical 2Cys-Peroxiredoxin (KDR23852.1) | *Z. nevadensis* |
| *RsPRX6* | comp804234_c2_seq1 | FX983169 | 1Cys-Peroxiredoxin (KDR15106.1) | *Z. nevadensis* |
| *RsGPX* | comp807654_c0_seq1 | FX983170 | Glutathione peroxidase 6-like (KDR10349.1) | *Z. nevadensis* |
| *RsPHGPX* | comp780877_c0_seq2 | FX983171 | Phospholipid hydroperoxide glutathione peroxidase (KDR22003.1) | *Z. nevadensis* |
| *RsGAPDH* | comp666388_c0_seq2 | FX983172 | Glyceraldehyde 3-phosphate dehydrogenase (KDR24072.1) | *Z. nevadensis* |
